# Cuproptosis-Associated lncRNA Gene Signature Establishes New Prognostic Profile and Predicts Immunotherapy Response in Endometrial Carcinoma

**DOI:** 10.1007/s10528-023-10574-8

**Published:** 2023-12-18

**Authors:** Xi-Ya Jiang, Jing-Jing Hu, Rui Wang, Wei-Yu Zhang, Qin-Qin Jin, Yin-Ting Yang, Jie Mei, Lin Hong, Hui Yao, Feng Tao, Jie-Jie Li, Yu Liu, Li Zhang, Shun-Xia Chen, Guo Chen, Yang Song, Shu-Guang Zhou

**Affiliations:** 1 Department of Gynecology and Obstetrics, Maternity and Child Healthcare Hospital Affiliated to Anhui Medical University, Anhui Province Maternity and Child Healthcare Hospital, Hefei, 230001 Anhui China; 2 Department of Gynecology and Obstetrics, The Fifth Clinical College of Anhui Medical University, Hefei, 230032 Anhui China; 3 Department of Reproduction, The First Affiliated Hospital of Anhui Medical University, Hefei, 230032 Anhui China; 4 Department of Clinical Laboratory, Anhui Province Maternity and Child Healthcare Hospital, Hefei, 230001 Anhui China; 5 Department of Pain, The First Affiliated Hospital of Anhui Medical University, Hefei, 230032 Anhui China

**Keywords:** Cuproptosis, Long non-coding RNA, Endometrial cancer, Prognostic signature, Immune landscape

## Abstract

Uterine corpus endometrial carcinoma (UCEC), a prevalent kind of cancerous tumor in female reproductive system that has a dismal prognosis in women worldwide. Given the very limited studies of cuproptosis-related lncRNAs (CRLs) in UCEC. Our purpose was to construct a prognostic profile based on CRLs and explore its assess prognostic value in UCEC victims and its correlation with the immunological microenvironment.Methods: 554 UCEC tumor samples and 23 normal samples’ RNA-seq statistics and clinical details were compiled from data in the TCGA database. CRLs were obtained using Pearson correlation analysis. Using LASSO Cox regression, multivariate Cox regression, and univariate Cox regression analysis, six CRLs are confirmed to develop a risk prediction model at last.We identified two main molecular subtypes and observed that multilayer CRLs modifications were related to patient clinicopathological features, prognosis, and tumor microenvironment (TME) cell infiltration characteristics, and then we verified the prognostic hallmark of UCEC and examined its immunological landscape.Finally, using qRT-PCR, model hub genes’ expression patterns were confirmed. Results: A unique CRL signature was established by the combination of six differently expressed CRLs that were highly linked with the prognosis of UCEC patients. According to their CRLs signatures, the patients were divided into two groups: the low-risk and the high-risk groups. Compared to individuals at high risk, patients at low risk had higher survival rates (*p* < 0.001). Additionally, Cox regression reveals that the profiles of lncRNAs linked to cuproptosis may independently predict prognosis in UCEC patients. The 1-, 3-, and 5-year risks’ respective receiver operating characteristics (ROC) exhibited AUC values of 0.778, 0.810, and 0.854. Likewise, the signature could predict survival in different groups based on factors like stage, age, and grade, among others. Further investigation revealed differences between the different risk score groups in terms of drug sensitivity,immune cell infiltration,tumor mutation burden (TMB) score and microsatellite instability (MSI) score. Compared to the group of high risk, the low-risk group had greater rates of TMB and MSI. Results from qRT-PCR revealed that in UCEC vs normal tissues, AC026202.2, NRAV, AC079466.2, and AC090617.5 were upregulated,while LINC01545 and AL450384.1 were downregulated. Conclusions: Our research clarified the relationship between CRLs signature and the immunological profile and prognosis of UCEC.This signature will establish the framework for future investigations into the endometrial cancer CRLs mechanism as well as the exploitation of new diagnostic tools and new therapeutic.

**Supplementary Information:**

The online version contains supplementary material available at 10.1007/s10528-023-10574-8.

## Introduction

UCEC occurs as the second common female reproductive system tumors globally. According to the GLOBOCAN database, 417,367 new UCEC cases and 97,370 deaths have occurred worldwide by 2020 (Bray et al. 2018). Postmenopausal women constitute the predominant demographic affected by endometrial cancer. Presently, the incidence of this condition exhibits a yearly rise, which can be attributed to the aging population and the escalating prevalence of obesity among women (Gu et al. 2021). It is thought that as many as 25% of cases were also found before menopause, and even if UCEC patients with early stage accepted surgical therapy got good prognosis, however, for patients with advanced stages or recurrent endometrial cancer, 5-year survival rates were only approximately 17% (Siegel et al. 2021).A lower five-year survival rate is indicated that these present approaches to risk and prognosis prediction based on the clinicopathologic characteristics of patients may be insufficient. As a result, it is of the utmost importance to locate new biomarkers to construct a risk prediction model for UCEC.

Copper is an important trace element because it participates in many metabolic reactions that take place within the human body. However,copper has dual effects on metabolic pathways in all species,excess intracellular copper is poisonous and can kill cells. Cuproptosis, which is thought to be a copper-triggered mechanism of mitochondrial cell death, has recently come into focus.Related studies suggest that compared to healthy populations, cancer patients had greater serum and tumor tissue copper levels (Blockhuys et al. 2017; Ge et al. 2022; Ishida et al. 2013). Intracellular copper levels could impact on the initiation and progression of cancer, and copper overload could cause cytotoxicity (Tchounwou et al. 2008).Copper dysregulation plays a role in the beginning and development of diseases like cancer. Several types of cancer have been found to have increased levels of copper in malignant tissues, including breast, lung, stomach, ovarian, cervical, and leukemia (Denoyer et al. 2015; Saleh et al. 2020).Cuproptosis is a mechanism of neuronal death that differs from previous recognized procedures (such as pyroptosis, apoptosis, and iron death). Cell proliferation, angiogenesis, and metastasis, the three main aspects of cancer progression are all influenced by copper (Hanahan and Weinberg 2011).Furthermore, it has been demonstrated that copper is capable of adhering directly to the lipoacylated elements of the TCA cycle, eventually leading to toxic protein dilatation and death of cells (Shimada et al. 2018; Tsvetkov et al. 2022). Consequently, it is thought that one new therapeutic strategy for killing cancer cells is to increase the buildup of intracellular cancer (Ge et al. 2022).There is convincing evidence linking copper levels to endometrial cancer (Chen 2022). The mechanism states that in UCEC patients, identifying the regulators of the unique type of cell death is essential.

Long non-coding RNAs (lncRNAs) is a term used to describe non-coding RNAs that exceed 200 nucleotides in length (Xiao et al. 2018).Cell- and tissue-specific expression of lncRNAs makes them good indicators for ongoing biological events. Several malignancies express lncRNAs differently and have varying clinical outcomes (Choudhari et al. 2020).Besides,lncRNA was found to be a key role in regulating tumor initiation and metastasis (Du et al. 2020). Even though studies on lncRNA in carcinoma are expanding, less is known about their function in the development of UCEC.

This study integrated bioinformatics analysis and experimental techniques to develop and evaluate the predictive characteristics of long non-coding RNAs (lncRNAs) associated with cuproptosis, a kind of cell death induced by copper.

We have also devised nomograms for the purpose of forecasting the clinical outcomes of patients. It has also been investigated whether risk scores and immune cells, mutational burden, and clinical therapeutic treatment sensitivity are correlated.In addition, an investigation was conducted into the molecular and functional pathways that exhibited enrichment among distinct risk populations.In conclusion, the prognostic characteristics of CRLs may be a reliable new predictor of prognosis in patients with UCEC and serve as a guide for treatment decisions.

## Materials and Methods

### Data Gathering and Preliminary Data Processing

Informations on somatic mutations, clinical characteristics, and gene expression profiles for endometrial cancer were obtained from the TCGA’s official website (https://portal.gdc.cancer.gov/). Then we used Perl to annotate the UCEC dataset and convert them into genes that code for protein and lncRNA. After eliminating samples missing complete lncRNA expression levels and clinical data, 577 samples (23 normal and 554 tumor) were included in the follow-up study.Using the genecard database and earlier studies, 19 cuproptosis-related genes were discovered (Liu et al. 2022).Following this, we used the R “limma” tool to figure out the relationship between genes related to cuproptosis and lncRNAs, Pearson Correlation analysis (|R|> 0.4, *p* < 0.001) (Schober et al. 2018), Overall, 1,136 lncRNA expressions associated with cuproptosis were found.To identify CRLs linked to UCEC patients’ prognosis, we used univariate Cox regression analysis.

### Creating and Verifying the Prognosis Risk Model Based on CRLs

To establish the predictive model, using a 1:1 ratio, We randomly split the total group into two groups: a training group and a validation group. After the Chi-square test and *T*-test calculations, there was no statistically discernible change in the clinicopathological features between two categories (*P* > 0.05). The training group was submitted to lasso-Cox regression analysis and multivariate Cox regression analysis( Friedman et al. 2010), to establish a prognostic risk score model which can forecast the OS of patients who provide UCEC samples. The prognostic risk score regression equation is: Risk score = (Expi × *β*i) (*β*: model gene coefficient; Exp: expression level of model gene). Then we divided the samples into high-risk and low-risk categories using the midpoint risk score cut-off value, and we created Kaplan–Meier survival curves using the R package “survival” to compare the survival rates of two groups. Using the “timeROC” R package, time-dependent receiver operating characteristic (tROC) curves were developed to assess the sensitivity and specificity of characteristics to predict 1-, 3-, and 5-year prognosis for UCEC patients (Kamarudin et al. 2017).To investigate whether the risk score was a distinctive prognostic marker, Cox regression tests with single and multiple variables were performed. Results showed that the risk factor was a distinct predictor from other clinical variables.

### Analysis of the Clinical Parameters in Stratified Fashion

In order to make clear how risk model scores and clinical characteristics relate to one another and determine whether the risk prognostic feature is applicable to different clinical subgroups, Age, Grade, and AJCC stage were used to build an analysis that is stratified depending on clinicopathological characteristics. Following survival analysis of all samples by subgroup showed that patients at high risk exhibited a significantly worse prognosis at >  = 65 or < 65 years, tumor stage I/II or III/IV, and tumor grade I/II or III/IV (*P* < 0.001).

### Establishing and Evaluating the Nomogram

Using the R packages “rms” and “regplot” we generated a nomogram based on the six-CRLs signature, which included the signature, age, and stage details.Based on the nomogram, we estimated the prognosis of UCEC sufferers at 1, 3, and 5 years.By plotting the calibration curves, we assessed the reliability and accuracy of the nomogram.

### Pathway Enrichment Analysis and PCA

The distribution of various patient categories was analyzed using the PCA method. In contrast to all CRLs, or whole-gene sets, we discovered that the novel CRLs signature could more clearly separate high and low risk patients. To gain a deeper understanding of the biological functions and pathways of DEGs in the two risk score groups, we performed GO and KEGG enrichment analyses (Shen et al. 2019, 2020; Shi et al. 2021). Subsequently, based on risk levels, we progressed the GSEA enrichment analysis of various risk categories, further intuitive comparison between tow risk groups the extensive enrichment pathway differences.

### Association Between Risk Rating Values and Immune Cell Infiltration

Based on our input expression matrix and immune cell marker gene set, ssGSEA can transform each sample’s gene expression profile from TCGA RNA sequencing data into the enrichment profile of a gene set, and utilize the enrichment score to figure out the level of immunological infiltration. In order to forecast, based on the CRL signature, the proportional amounts of tumor-infiltrating immune cells (TIICs) in the two risk categories. Using the ssGSEA methodology, we assessed the abundance and related functions of the 23 main types of immune cells, the relationship between risk level and immunological function was then further researched.

### Mutation Patterns in Different Risk Categories

To demonstrate how UCEC causes somatic gene alterations in samples with different risk scores, we assessed the frequency of tumor mutations in the two categories. Using the “maftools” R package, the amount of somatic non-sense point mutations in each sample has been evaluated (Mayakonda et al. 2018) After that, using the “ggpubr” R package and a Kaplan–Meier survival analysis, compare the survival traits of UCEC patients in various TMB groups.

### Sensitivity Testing for Drugs

To identify prospective chemotherapeutic medications that might be applied in UCEC therapy, the value of frequently used chemotherapeutic agents in IC50 for UCEC was projected using the “pRRophetic” R package (Geeleher et al. 2014).

### The qRT-PCR and Cell Culture

In this study, two human endometrial cell lines HEEC (endometrial epithelial cell line), HESC (endometrial stromal cell line) and three UCEC cell lines (HEC-1A, KLE, Ishikawa) were used. All cells were purchased through the American Type Culture Collection (ATCC). These cells were cultured in F-12, McCoy’s 5A or Leibovitz’s L-15 medium (Gibco BRL, USA). Total cellular RNA was extracted from cell lines using 10% fetal calf serum (Gibco BRL, USA) using a standard protocol based total RNA extraction reagent (10,606 ES 60, Yeasen) at 37 °C, 95% humidity, and 5% CO_2_ cell incubator. The obtained RNA was then used for cDNA synthesis in the cDNA synthesis kit (11,139 ES 10, Yeasen). Gene expression was quantified using SYBR Green Yeasen & 11,201 ES 30 Yeasen, 3, and using the 2ΔΔCt method.For normalization, GAPDH is used as an internal standard. All primers used for qRT-PCR were synthesized by suborganisms (Shanghai). In Supplementary Table 2, a list of the primer sequences used in our research is provided.

### Statistical Analysis

All data analysis were carried out using the R program (version4.2.2). The Chi-square test was used to compare the differences of the clinical characteristics of the three subtypes (Guo et al. 2023). Gene expression correlation was determined using Pearson correlation analysis. Using univariate, Lasso, and multivariate Cox regression analyses sorted CRLs and established predictive characteristics. Using the Kaplan–Meier and log-rank testing techniques, the rate of survival behind the two distinct groups were compared. Using the “time ROC” package, the receiver operating characteristic (ROC) and area under the curve(AUC) were used to assess the specificity and sensitivity of the risk score. A p value < 0.05 was considered statistically significant, and all tests were two-tailed.

## Results

### LncRNA Linked to Cuproptosis in UCEC Patients

**Fig. 1:**
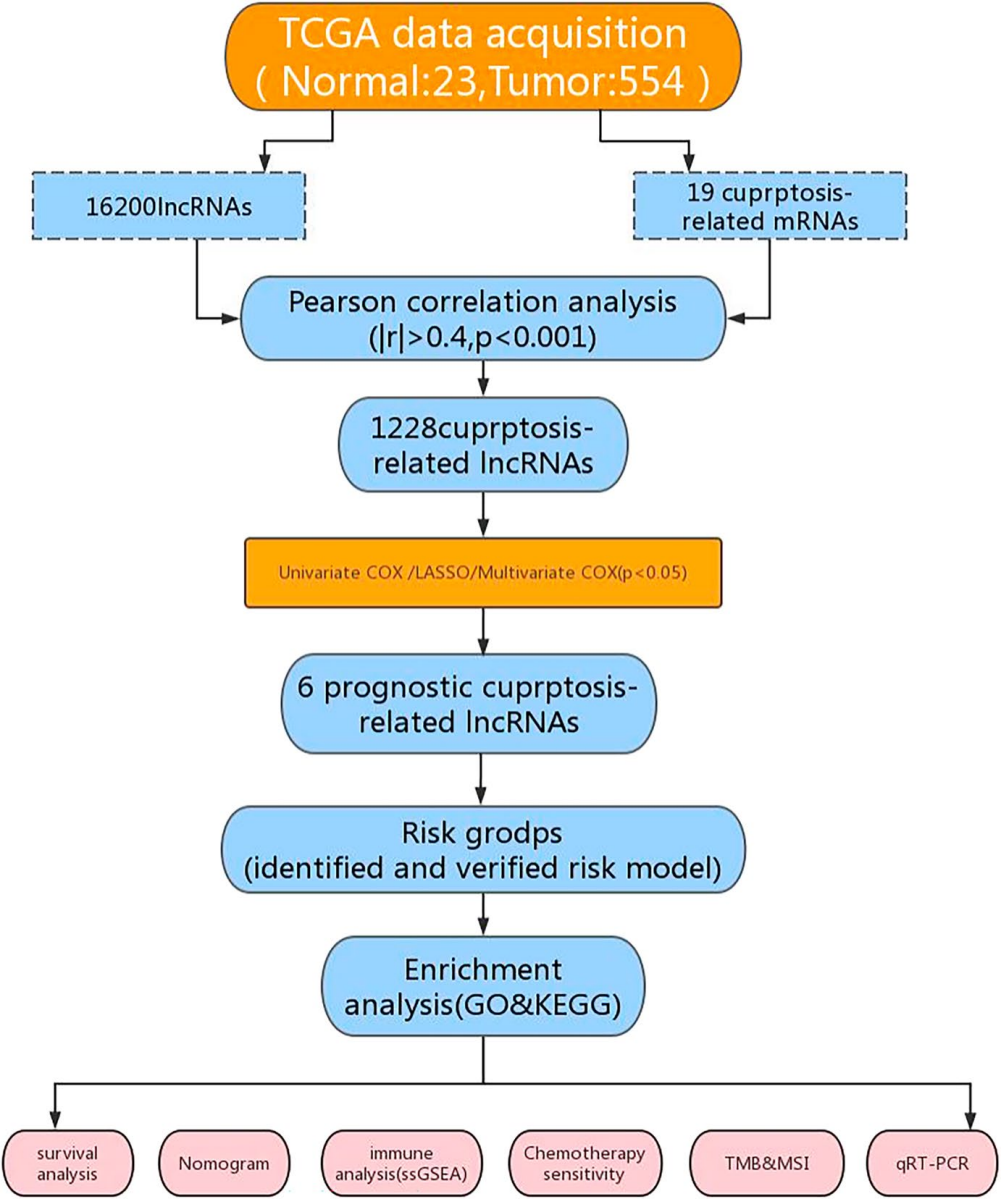
Experimental design flow chart

**Fig. 2:**
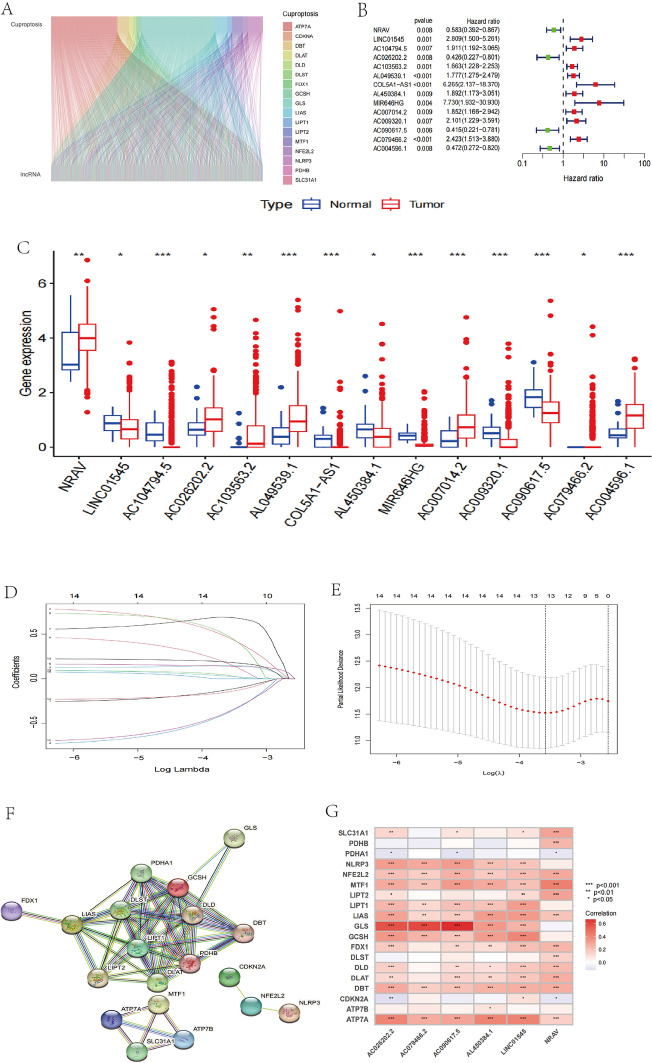
Identification of CRLs associated with UCEC prognosis. **A** Sankey plot was utilized to depict the co-expression network of lncRNAs associated to cuproptosis. **B** The forest plot of screening prognostic CRLs by univariate Cox regression analysis. **C**14 CRLs express separately in tumor and normal tissues. Represents an adjusted *p*-value (*p < 0.05, **p < 0.01, ***p < 0.001). **D** Distribution plot of the LASSO coefficient for the 14 CRLs. **E** LASSO regression model of CRLs. **F** The PPI network of cuproptosis-related genes. **G** Heatmap of the correlation between cuproptosis-related genes and lncRNAs

Figure 1 summarizes the study’s procedure.First, we obtained information on 554 UCEC samples and 23 routine samples from the TCGA database. In the ensembl gene annotation file from this dataset, 16,200 lncRNAs in total were found. By Pearson correlation analysis (|Pearson R |> 0.4 and *p* < 0.001), 1228 lncRNAs were chosen as CRLs. Using a sankey diagram, the 1228 lncRNAs and mRNAs related to cuproptosis were put together in a co-expression network (Fig. 2A).We integrated survival data from 543 patients with CRLs expression data, excluding 23 non-tumor sam ples and 11 samples that insufficient clinical baseline informations. The patients’ complete clinicopathological information is displayed in Supplementary Table 2. Following that, a random procedure was used to divide the TCGA dataset into the testing-set (*n* = 271) and the training-set (*n* = 272). In order to learn more about the predictive usefulness of the CRLs, we performed a univariate Cox regression analysis on the UCEC patients in the training-set.14 lncRNAs were discovered to be significantly linked to UCEC OS (P < 0.05). Significant variations in these CRLs expression for both tumor and normal tissues can be seen in bar graphs (Fig. 2B). The forest plot depicts the risk ratios and confidence intervals for the 14 prognosis-related lncRNAs studied, demonstrating that the vast majority of these genes are risk genes (Fig. 2C). The signature was simplified using LASSO-Cox regression analysis to improve prediction accuracy and prevent overfitting. 13 lncRNAs related to cuproptosis were found utilizing the minimal partial likelihood deviance (Figs. 2D, E). By using multivariable Cox regression analysis, six lncRNAs substantially linked to prognosis were found later on, namely LINC01545, AC02620.2, NRAV, AL450384.1, AC079466.2 and AC090617.5. Among these prognostic factors, LINC01545, AL450384.1, and AC079466.2 were discovered as risk factors (HR > 1), while AC026202, NRAV, and AC090617.5 were protective factors (HR < 1).Utilizing the Cox equations and increased expression of the six potential lncRNAs, the risk score (RS) for people with UCEC was created (Fig. 2F): RS = (0.49482*AC079466.2expression) + (− 0.43215*NRAVexpression) + (0.91419*LINC01545expression) + (0.76644*AL450384.1expression) + (− 0.78338*AC090617.5expression) + (− 0.84984*AC026202.2expression).The correlation between the six potential prognostic lncRNAs and CRLs was demonstrated by a correlation heatmap (Fig. 2G).

### Evaluation of the Prognostic Models Across the Various Groups

**Fig. 3:**
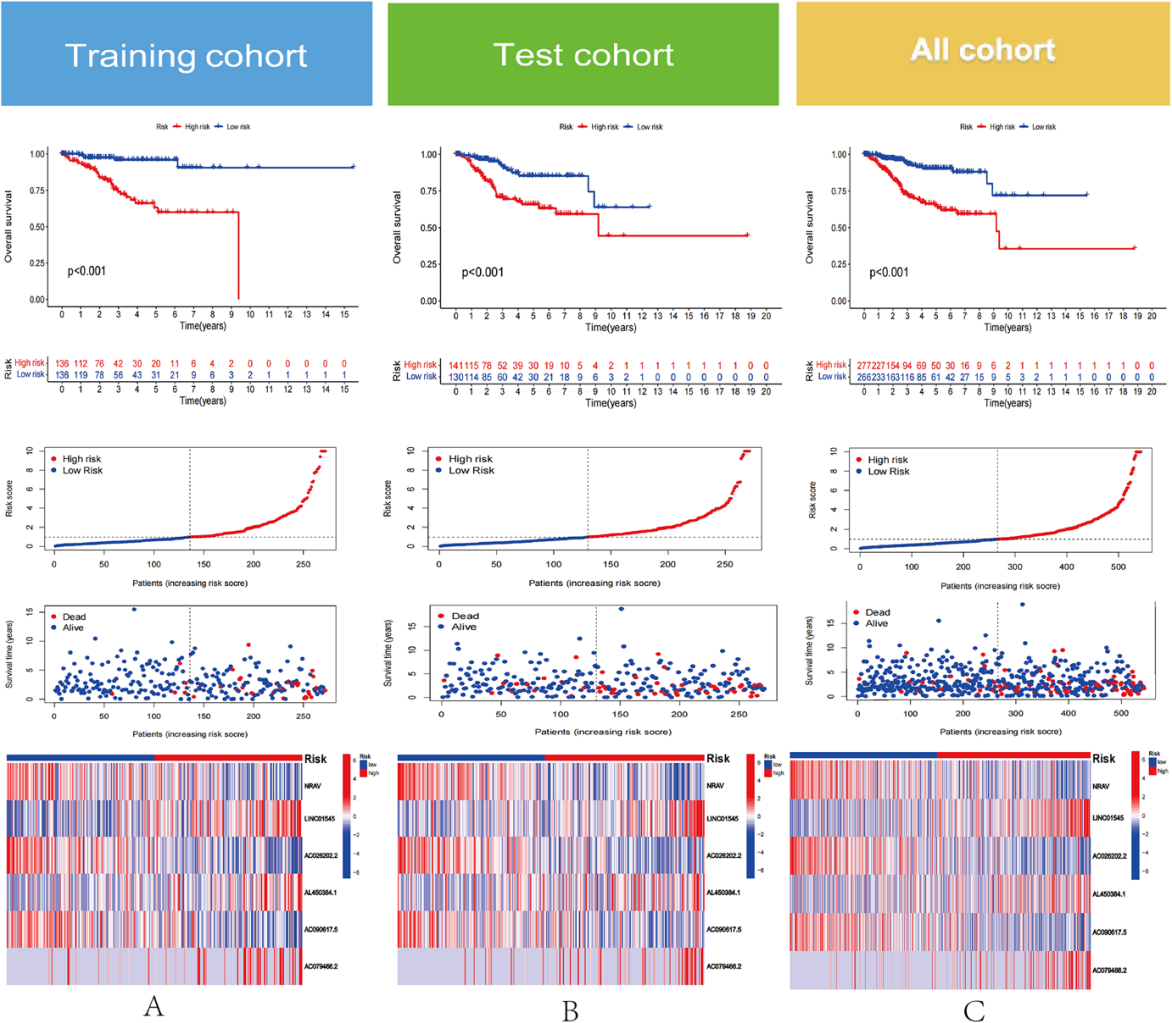
Development and validation of the prognostic CRLs signature. **A** A Kaplan–Meier survival curve analysis reveals a correlation of different risk score with survival time (top).Scatter plots were employed to display the connection between the risk score and survival time(middle).The association between the prognostic feature and the CRLs are illustrated by the heatmap (bottom). **B**, **C** The test cohort and the complete cohort underwent the identical procedure

We categorized all samples into different categories based on the median risk score value of UCEC. Indicator of Kaplan–Meier survival showed that in the training cohort, test cohort, and overall cohort, higher risk score UCEC sufferers exhibited a worse OS. The prognostic feature model successfully predicted prognosis in 3 separate cohorts, according to a time-dependent ROC curve (Training cohort 1-year AUC = 0.778, 3-years AUC = 0.810,5-years AUC = 0.854; Test cohort 1-year AUC = 0.673,3-years AUC = 0.700,5-years AUC = 0.643; All cohort 1 year AUC = 0.724,3-years AUC = 0.745,5-years AUC = 0.736).Different risk sets’ 1-, 3-, and 5-year AUC values were all greater than 0.6, demonstrating the model’s outstanding capacity to predict mortality across a range of sets (Fig. S3). Additionally, each group’s feature lncRNAs associated to the prognostic model are presented in a heat map. All of these evidence suggests that CRLs prognostic traits are accurate predictors of the outcome for UCEC patients (Fig. 3A–C).

### CRLs Can Serve as a Key Independent Predictor of Outcome

**Fig. 4:**
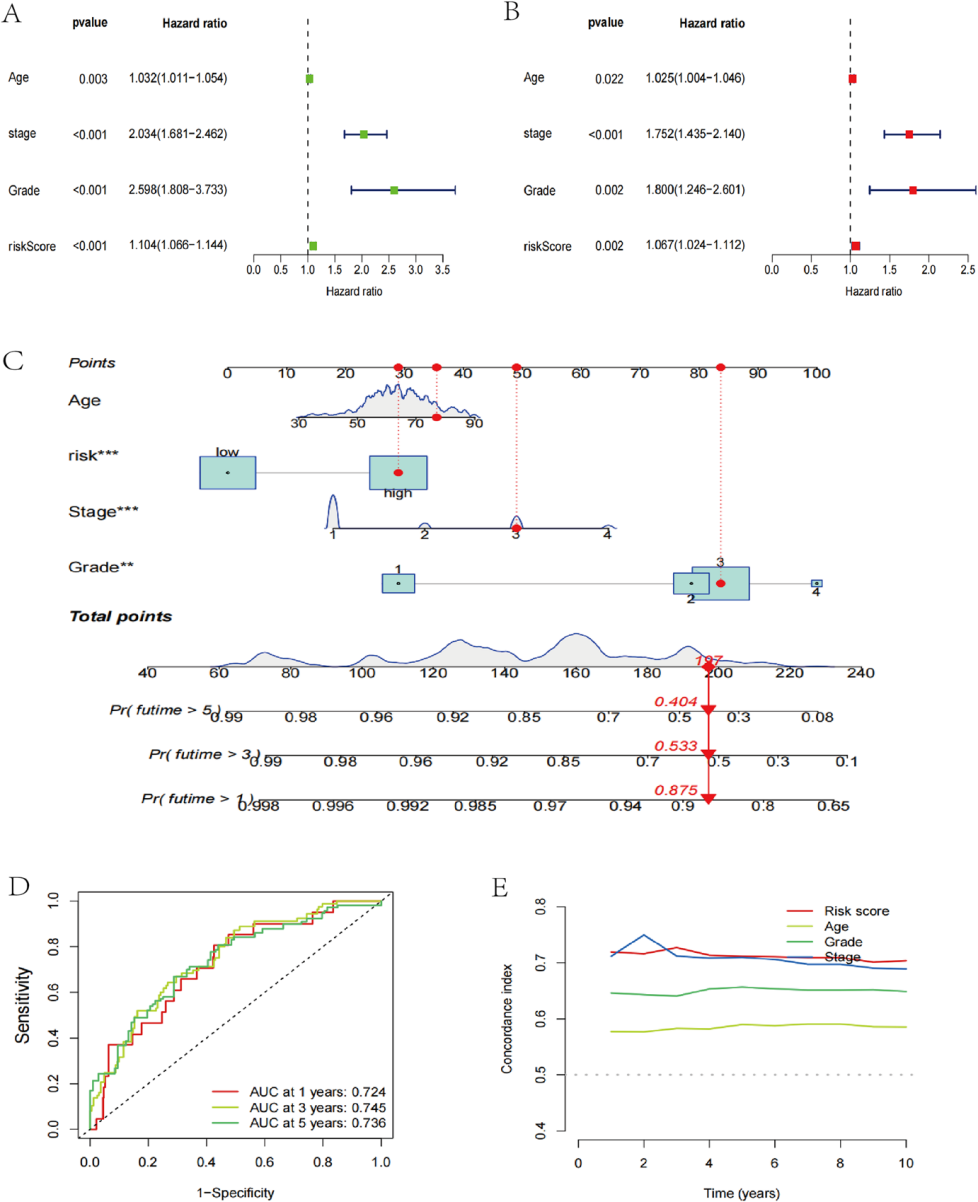
Assessing the risk model’s ability to predict. **A** A univariate Cox analysis of the OS and clinical traits for the risk score. **B** Multivariate Cox analysis of the OS and clinical factors for the risk score. **C** Nomogram validating the OS of patients with UCEC. **D** The calibration curve evaluated the consistency between the anticipated 1-, 3-, and 5-year survival rates and the actual OS rate. **E** ROC curves of risk scores and other clinical characteristics based on OS are shown. OS, overall survival; ROC, receiver operating characteristic; UCEC, Uterine corpus endometrial carcinoma. *means *p* < 0.05, **means *p* < 0.01, ***means *p* < 0.001

A single-variate Cox analysis, there existed a substantial correlation among OS and grade, stage, age, and risk score. In contrast, multivariate Cox regression analysis also showed a statistical association between OS and stage, grade, age, and risk score (*P* < 0.05). Therefore, prognostic indicators based on six CRLs were independently predictive of survival in UCEC patients (Fig. 4A, B).To further assess the predictive accuracy and clinical application, we created a nomogram with both prognostic features and conventional clinicopathological markers (Fig. 4C). The calibration figure showed great concordance between the estimated rates of 1-, 3-, and 5-year OS and predicted in patients with UCEC (Fig. 4D). Finally, we compared the ability of six CRLs features in assessing UCEC patients’ survival, and it shown that the risk score had the greatest C-index, demonstrating our accuracy, and outperformed other clinical traits (including pathological grade, stage, and age) (Fig. 4E).

### Risk Scores and Clinicopathological Variables Were Highly Correlated

**Fig. 5:**
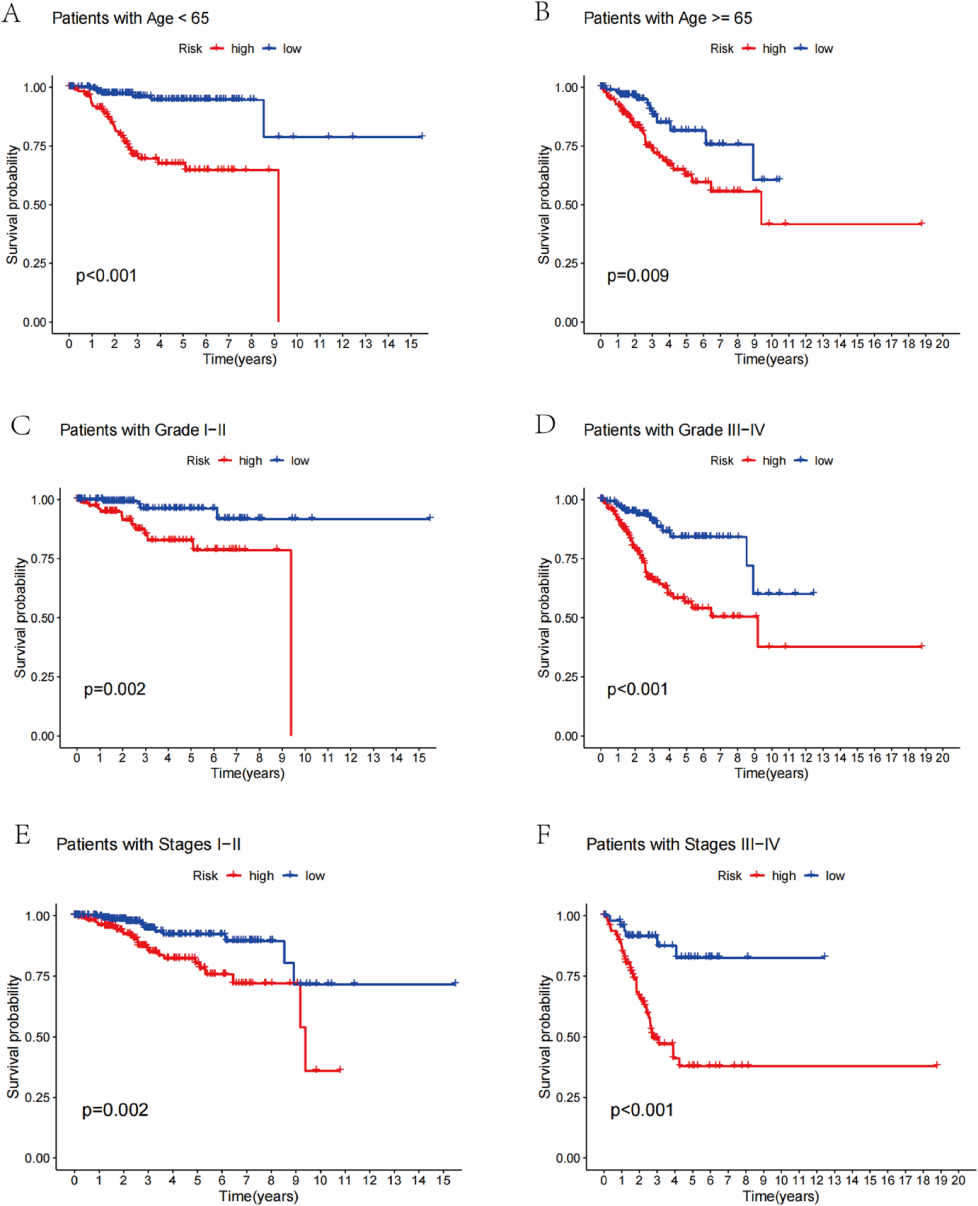
Kaplan Meier survival curves of risk subgroups in patients stratified by different clinicopathological factors. **A** Age < 65 years; **B** Age >  = 65 years; **C** Grades I–II; **D** Grades III-IV; **E** Stages I–II; **F** Stages III–IV

To determine whether the risk score can effectively forecast patient mortality, we stratified the essential clinicopathological characteristics, such as age, stage, and grade. Compared with patients with low-risk scores, high-risk score patients in the younger subgroup (< = 65 years, *P* = 0.009) (Fig. 5A), advanced age subgroup ( > 65 years, *P* = 0.001) (Fig. 5B), stage I–II (*P* = 0.002) (Fig. 5C), stage III–IV (*P* < 0.001) (Fig. 5D), and low-grade group (Grade I–II, *P* = 0.002) (Fig. 5E), high-grade group (Grade III–IV, *P* < 0.001) (Fig. 5F) all had a poor prognosis. This clearly illustrates that the six CRLs we investigated are each extremely reliable indications of the prognosis for endometrial cancer patients with a variety of clinical manifestations.

### Functional Enrichment Analysis and PCA

**Fig. 6:**
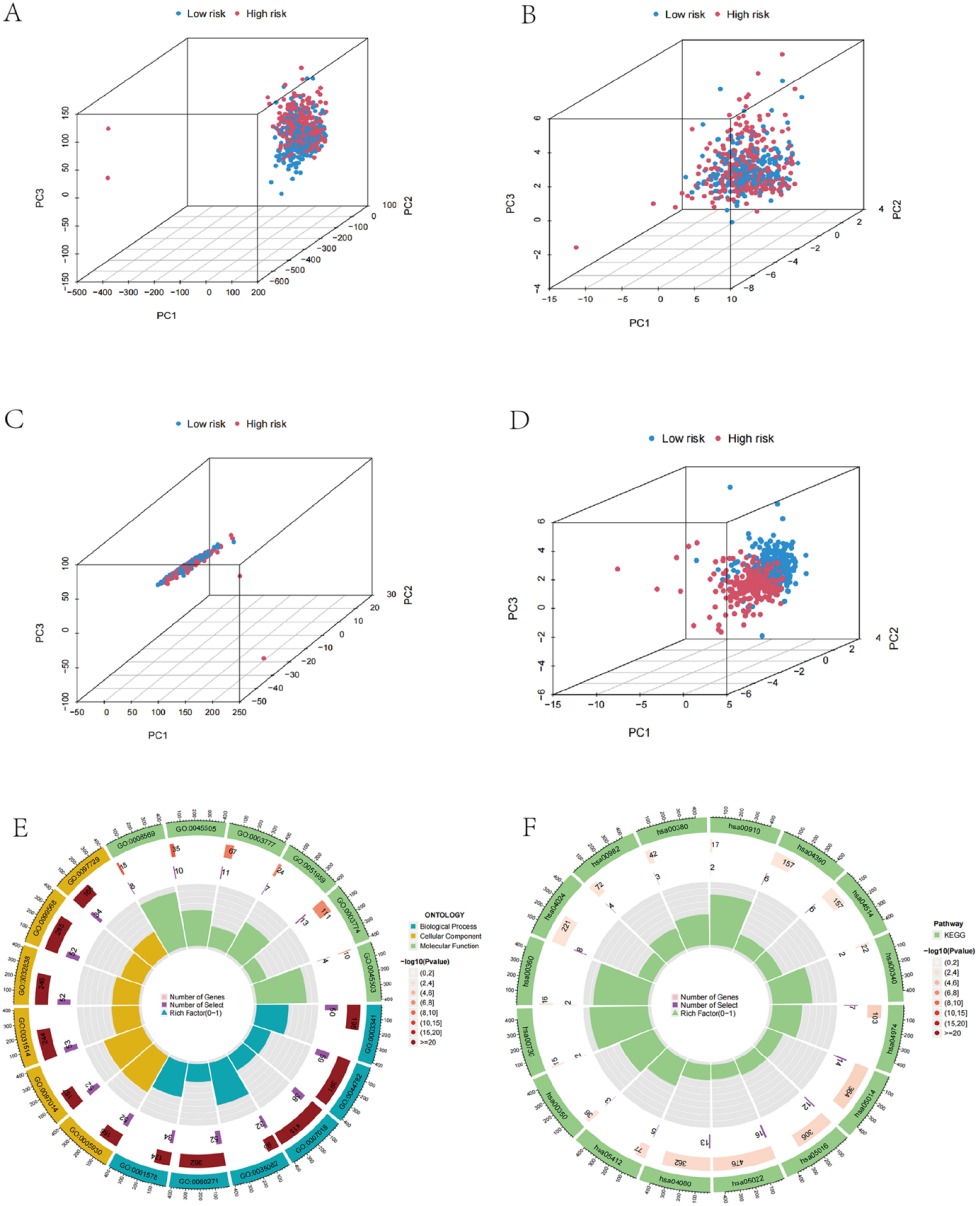
PCA analysis and enrichment analysis. **A**–**D** PCA plots for all risk genes, risk score, CRLs, and cuproptosis-related coding genes. **E** The circle graph demonstrated that the top GO signaling pathways involved biological processes in BP, MF, and CC. **F** KEGG analysis of differential genes in the prognostic model’s high- and low-risk groups (circle plot)

To learn more regarding the level of gene epigenetic of CRLs, we investigated the overall distribution of endometrial cancer patients using PCA analysis and various typing techniques. The results indicated that six CRLs features, rather than the entire CRLs, cuproptosis-associated mRNAs, or the whole genome could more accurately identify between patients at low and high risk (Fig. 6A–D). To gain insight into the discrepancies in biochemical functions and signaling molecules between the different risk organizations, we identified differential genes with log^2^|FC |> 1 and FDR < 0.05 for the sake of GO and KEGG enrichment analysis and visualized the leading 15 outcomes.In GO analysis, the main biological process (Supplementary Table 3) in differential gene enrichment is microtubule − based movement, cilium organization, cilium assembly andcilium movement. The molecular function enrichment was mainly focused on the tubulin binding, microtubule motor activity and cytoskeletal motor activity, while the cellular components were mainly plasma membrane bounded cell projection cytoplasm, motile cilium,and cytoplasmic region (Fig. 6E).Furthermore, depending on KEGG analysis, the molecular pathways of neurodegeneration-multiple diseases, muscular atrophy-lateral sclerosis, and neuroactive ligand receptor interaction were considerably enriched (Fig. 6F).

**Fig. 7:**
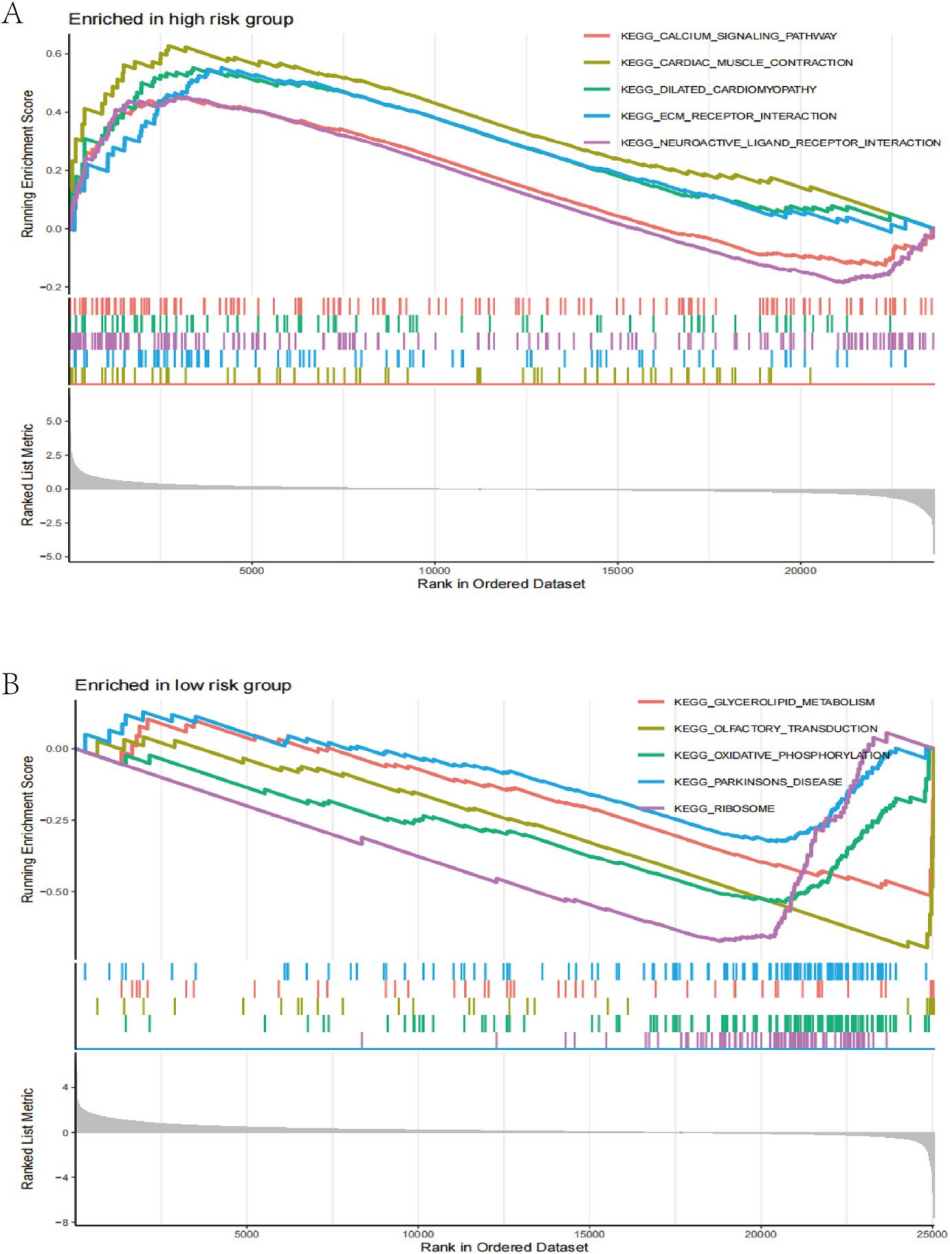
Gene set enrichment analysis (GSEA) for different risk subset. **A** GSEA of the notably enriched pathways in high-risk subset. **B** GSEA of significantly enriched pathways in low-risk subset

We subsequently analyzed the different risk score groups in biological function and pathways by GSEA analysis to further elucidate the variations in biological function between different risk groups.Findings indicated that the calcium signaling pathway, ECM-receptor interaction pathway,neuroactive ligand receptor interaction, heart muscle contraction and dilated cardiomyopathy were more prevalent in the high-risk subgroup (Fig. 7A). Parkinson’s disease, glycerolipid metabolism,oxidative phosphorylation, olfactory transduction, and the ribosome pathway were more prevalent in the low-risk subgroup(Fig. 7B).

### Different Risk Groups Have Distinct Immune Profiles

**Fig. 8:**
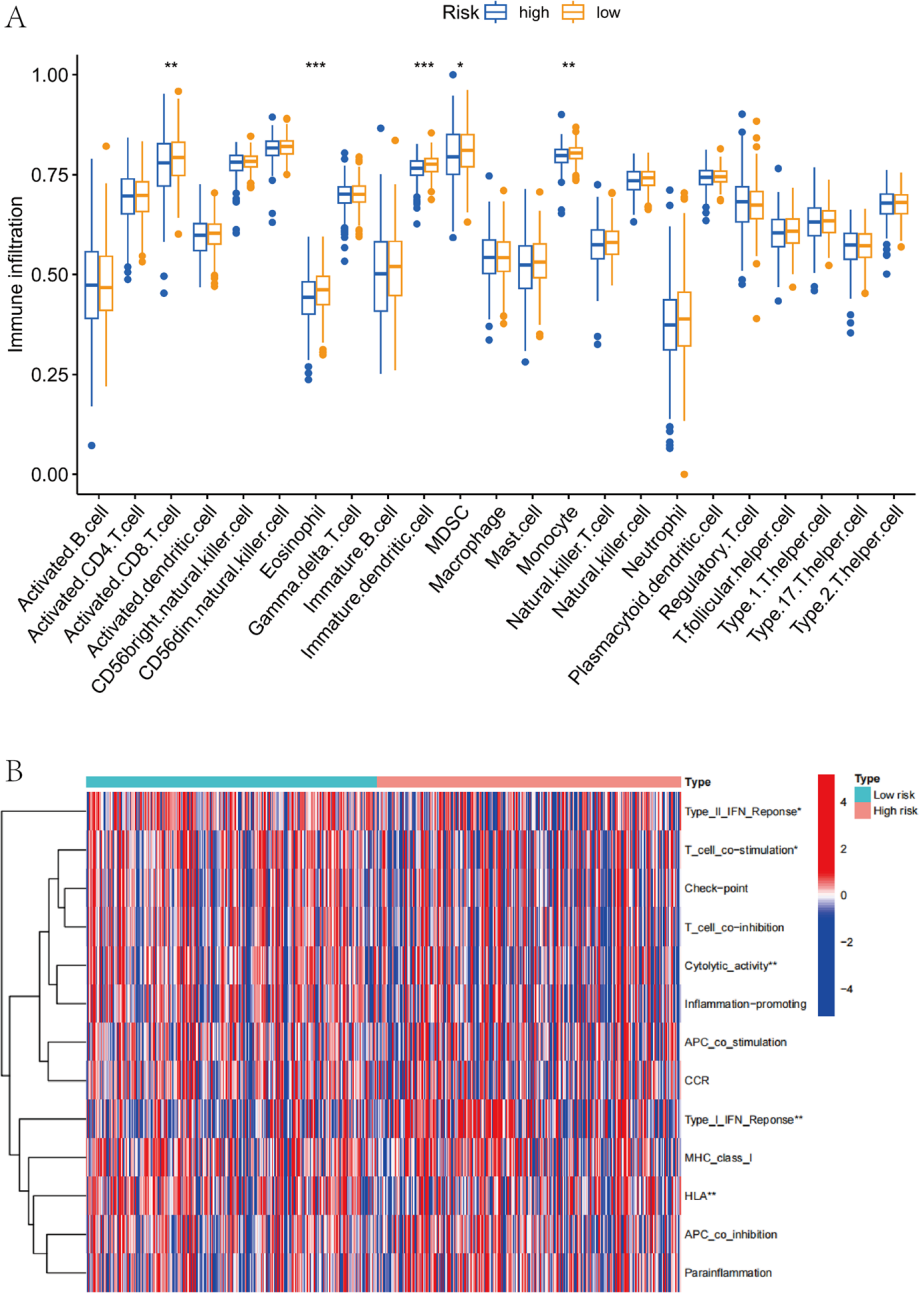
Immune cell infiltration features in the risk subgroups. **A** ssGSEA for the relationship between TIICs and related functions in various risk groups. **B** Heatmap using the ssGSEA approach to show the variations in immune-related functions between the two risk groups. *p < 0.05, **p < 0.01, and ***p < 0.001

We quantified the enrichment scores of the 23 immune cell subsets and their associated immune functions or immune pathways using ssGSEA aim to better explore the differences in immunological status among the various risk score groups. The findings demonstrated that many immune cell types showed substantial differences with low and high risk scores (*P* < 0.05, Fig. 8A), including activated CD8^+^ T cells, eosinophils, immature dendritic cells, MDSC, and monocytes. Additionally, the low-risk score group was more prevalent in Type II IFN Response, Cytolytic activity, T cell co-stimulation, and HLA (*P* < 0.05, Fig. 8B). To further assess the immune infiltration differences between the high and low risk groups,we validated our data correlated with immune cells by using the “TIMER” (http://timer.cistrome.org) analysis tool.Furthermore, we also used the CIBERSORT algorithm to predict immune cell infiltration in tumor samples(Thorsson et al. 2018), with a high overlap between the results of the different algorithms (Fig. S8A, B).For example, those for ssGSEA,CIBERSORT and TIMER multiple immune infiltrations indicated a link between CD8^ +^ T cell enrichment and low risk scores. Previous studies have also showed that the CD8 ^+^ T cells are crucial for protective immunity against intracellular infections and malignancies (Kurachi 2019), indicating that knowing the molecular mechanism of T cell depletion is essential for developing effective immunotherapeutic approaches.However, due to the diversity of algorithms and the difference of immune cell types, the results of each algorithm also are slightly difference.

**Fig. 9:**
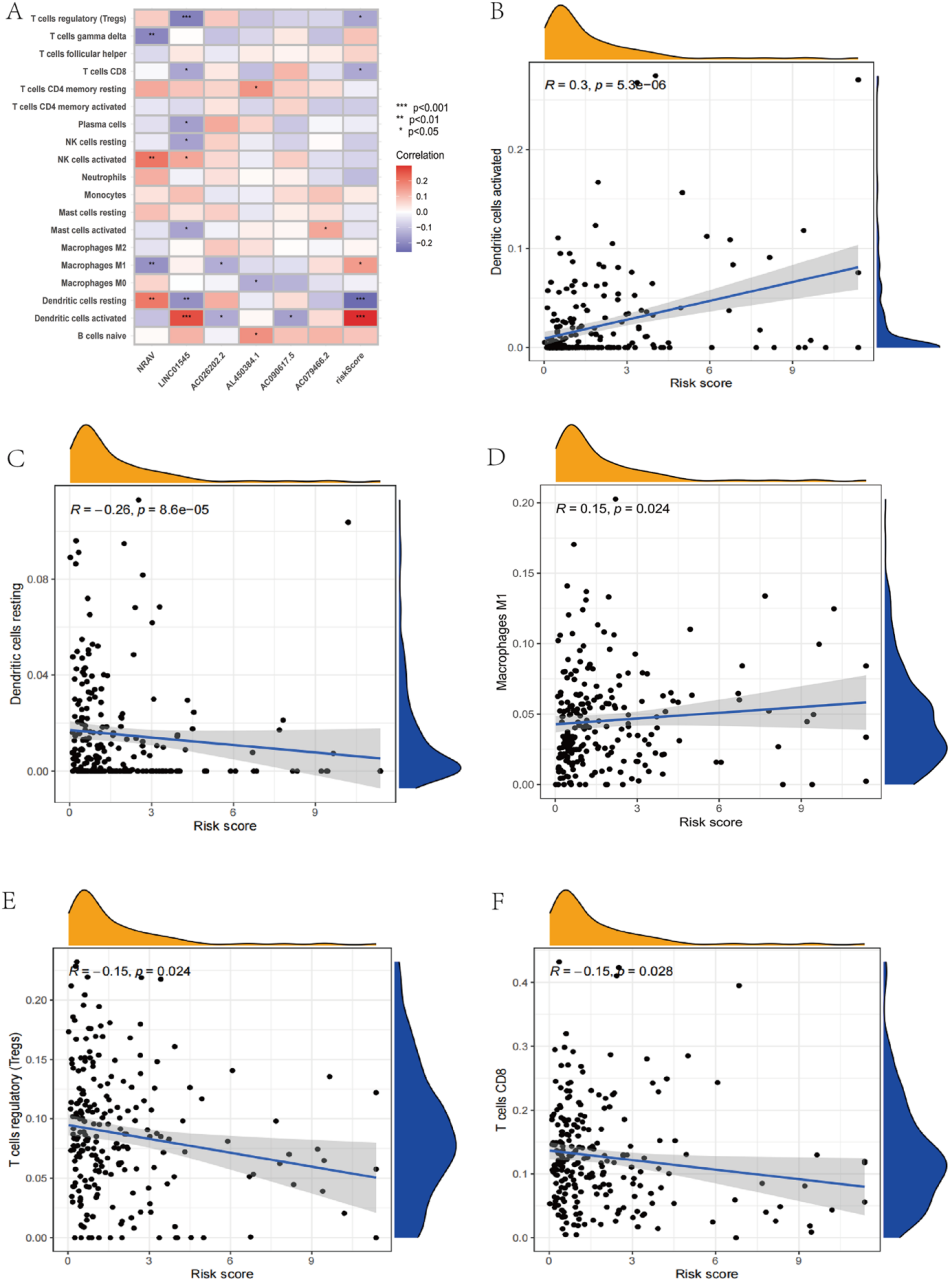
The relationships between the risk scores calculated by the 6-lncRNA signature and immune infiltration cells were evaluated. **A** A corrplot was employed to demonstrate the association between the 19 immune cells, risk ratings, and the six lncRNAs. **B**–**F** Risk assessments and the relationship between various tumor immune cells *p < 0.05, **p < 0.01, and ***p < 0.001

It is generally recognized that TIICs can influence the immunological microenvironment, which in turn can influence tumor formation. To further comprehend the connection between immune cell invasion and lncRNAs associated to cuproptosis.We created a heatmap to illustrate the connection between immune cells and lncRNAs relevant to cuprotosis (Fig. 9A), then demonstrate how the relationship between the risk index and immune infiltrating cells is statistically significant.Co-expression pattern among immune cells and the risk score based on cuproptosis-related lncRNAs prognosis characteristics were significantly correlated with the infiltration levels of resting DCs (*R* = − 0.26 *P* = 8.6e-05), activated DCs (*R* = 0.3, *P* = 5.3e-06), macrophages M_1_ (*R* = 0.15, *P* = 0.024), and CD8^+^T cells (*R* =− 0.15, *P* = 0.028), regulatory T cells (treg) (*R* = − 0.15, *P* = 0.024) (Fig. 9B-F). In conclusion, the immune cell invasion is linked to the cuproptosis-related lncRNAs of UCEC.

### Alteration Landscapes Among High- and Low-Risk Score Categories

**Fig. 10:**
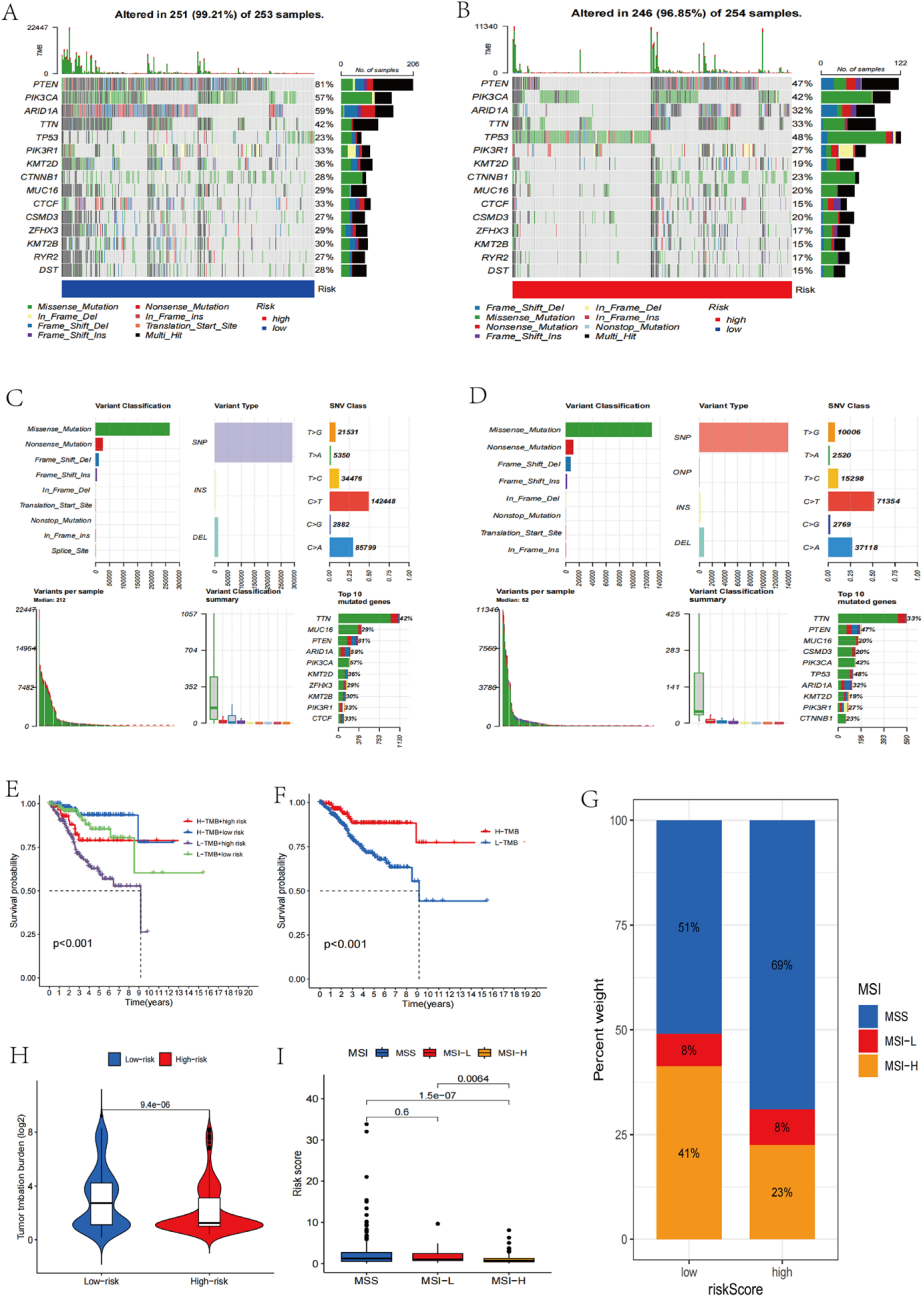
The mutational patterns and MSI of different populations at risk. **A**, **B** Somatic mutations between groups of people with different risk score. **C**, **D** The somatic mutation profiles of the high-risk group and low-risk group in UCEC patients. **E** H-TMB and L-TMB patient subsets’ Kaplan–Meier curves for OS. **F** The OS Kaplan–Meier curve for TMB plus risk. **G** The bar plot is used to show the relative frequency of the various risk groups among the low and high mutation groups. **H** TMB discrepancy between high-risk and low-risk individuals on a violin plot. **I** Boxplot displaying the risk scores for the MSI-H, MSI-L,and MSS

To learn more about the variations in tumor mutation status among the various risk categories, we assessed the tumor mutation burden within these groups. A significant variation in TMB status between the two groups was shown by the TMB analysis (Fig. 10A, B).When compared to the high-risk subset, the low-risk subset exhibited a larger percentage of mutations (Fig. 9G). We separated the UCEC samples into subsets with different mutations by the median TMB score. Patients with low mutation rates significantly outlived those with high mutation rates, the level of TBM load and patients’ overall survival rates were found to be correlated by a Kaplan–Meier analysis (Fig. 9E, F). In the examination of tumor mutations, the low-risk group’s tumor mutation rate reached 99.27%, which was higher than 96.85% in the high-risk group. Among those at low risk score group, the PTEN gene mutation was the most common (81%); Among those at high risk, the TP 53 gene mutation was more common (48%). In addition, missense mutations and single-nucleotide polymorphism mutations were most widespread in the two risk groups (C > T), followed by (C > A). (Fig. 10C, D) illustrates somatic mutation details. The MSI status of the tumor might be categorized into three categories: high (MSI-H), low (MSI-L), and stable (MSS). Our data demonstrate lower risk scores in individuals with MSI-H (high microsatellite instability), compared with two other low microsatellite instability phenotypes, such as MSI-L and MSS, in solid tumors, including endometrial cancer, CRC, and gastric cancer (Fig. 9H, I). These findings imply that endometrial cancer patients’ risk scores due to the six cuproptosis-related lncRNAs could present genomic stability.Considering the findings above, we concluded that risk score model has the ability to predict immunotherapy response.

### Sensitivity Testing for Drugs

**Fig. 11:**
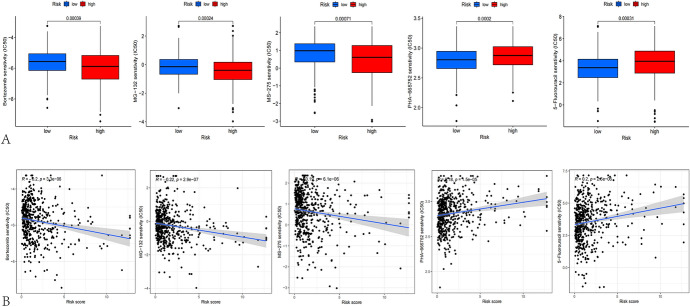
Drug sensitivity testing. **A** The IC50 of small molecule drugs in two risk populations, drug left to right Bortezomib, MG-132, MS-275 (Entinostat), PHA-665752,5-FU. **B** Correlation plots Bortezomib, MG-132, MS-275 (Entinostat), PHA-665752,5-FU.IC50, half of the maximum inhibitory concentration

Given the importance of chemotherapeutic drugs for UCEC, we further performed a sensitivity analysis of chemotherapeutic drugs in different groups and compared the IC50 values of sensitive drugs in patients with the two subtypes. Our findings indicated that low-risk score group had greater MG-132, MS-275 (Entinostat), and Bortezomib IC50 values, while the IC50 values of (5-FU) and PHA-665752 were higher in high-risk score group, which further demonstrated that the statistical significance of differences in commonly used chemotherapy medications divided into risk score categories, which is beneficial to provide a reference for screening sensitive chemotherapeutic drugs in people with diverse risk scores (Fig. 11A, B).

### Verification of lncRNA Expression Associated with Cuproptosis

**Fig. 12:**
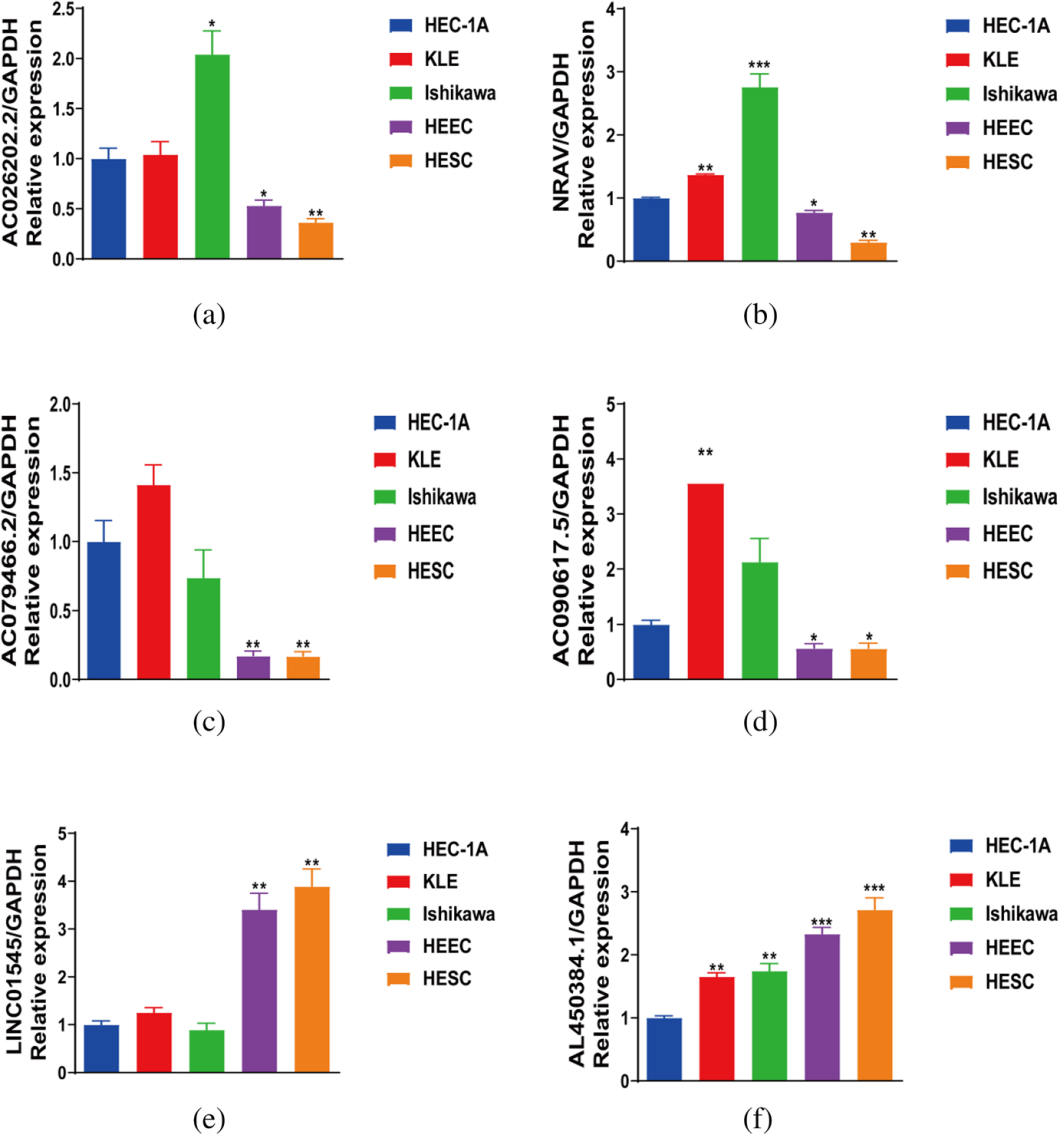
Verification of the expression level of the six CRLs in UCEC cell lines. **a**–**f** Relative expression of six CRLs in tumor cell lines (HEC-1A, KLE, Ishikawa) and normal cell lines (HEEC, HESC).The results of this study were all compared with the expression levels of the HEC-1A cell lines. *p < 0.05, **p < 0.01, and ***p < 0.001

To further validate the results of the bioinformatics analysis described above, qRT-PCR tests were conducted on various endometrial cancer cell lines. Our findings revealed that the expression of AC079466.2, AC090617.5, AC026202.2, and NRAV was markedly increased in the three common endometrial cancer cell lines (HEC-1A, KLE,and Ishikawa cell lines) compared to the human normal endometrial epithelial (HEEC) and endometrial stromal cell line (HESC) (Fig. 12a–d).In addition, our experimental results further showed that the expression level of LINC01545 and AL450384.1 was up-regulated in non-endometrial cancer cell lines, and between the HEEC and HESC cell lines, there was no statistically significant change in the expression of these two genes (Fig. 12e, f). The sequencing process of the target lncRNA was performed at the multicellular line level, so we have every reason to believe in the reliability of the results of this experiment. Overall, the results of this experiment matched our bioinformatics analysis, and further verified validated the accuracy of our risk measurement based on the six lncRNA associated with cuproptosis. For additional assurance that the target gene is expressed at the same level across most cell lines,we further used the cell line database CCLE (Since The Cancer Cell Line Encyclopedia), the results showed that six lncRNAs were expressed in 28 cervical cancer cell lines and the expression trend was similar to the biocredit analysis (Fig. S12A, B),but there were also differences in expression between the different cell lines.We examined possible causes. Tissues consist of various cells, and lncRNA expression varies among cell lines. The expression in tissues follows the same pattern as in all cells. The cell line we chose may have different lncRNA expression. But what is more noteworthy, we found that only included endometrial cancer cell lines and distant metastatic cell lines lacked non-tumor lines in the CCLE database,it is therefore impossible to contrast the differences between the tumor and non-tumor cell lines.

## Discussion

UCEC is best commonly diagnosed cancer of the female reproductive system, with the aging population in recent years and the decreasing fertility rate (Tsilidis et al. 2015; Wang et al. 2022a, b), the increasing incidence of endometrial cancer. The research found that the distant metastasis of endometrial cancer patients 5-year survival rate is only 16% (Chen et al. 2021). Although treatment may continue, due to tumor heterogeneity, even if UCEC patients have similar clinical characteristics and treatment, their prognoses can vary greatly. This shows that clinical stage and pathological classification alone are not enough to determine a patient’s prognosis. Early identification of endometrial cancer is difficult without unambiguous signs. Create accurate prognostic indicators to revive the UCEC prognostic forecast, stratify patients by risk profile, and predict drug susceptibility to give patients customized treatment options is essential.

The acknowledgment of RNA regulation as a pivotal aspect in the process of gene expression and the emergence of phenotypic intricacy originated from novel techniques and biological understandings that were established during the period of the 1970s–1980s (Sharp 2009).Extensive investigations into the transcriptome of mammals have revealed that the quantity and diversity of long non-coding RNAs (lncRNAs) surpass those of messenger RNAs (mRNAs) that encode proteins. Views on eukaryotic gene expression have changed recently due to the discovery that RNA can produce a great deal of variation (Licatalosi and Darnell 2010).When whole genome sequencing became possible, it was discovered that 99.7% of the coding regions in the human and chimpanzee genomes are the same (Calarco et al. 2007). This finding led to the novel theory that RNA is the fundamental component of biological complexity, which includes variations in cell type and function (Licatalosi and Darnell 2010).According to recent studies, cuproptosis is the most new method of cell death, and its associated lncRNAs significantly influence the growth of malignancies, inflammation, the immune system, drug resistance, and other factors (Wang et al. 2022a, b), and existing studies have confirmed that it can indeed be utilized as a standalone predictor of prognosis for cervical cancer, liver cancer, breast cancer, etc. It is reasonable to believe that cuproptosis-related lncRNAs is a prospective therapeutic target and prognostic marker for UCEC.Thus, this study examined cuproptosis-related lncRNAs to predict UCEC patient survival.

This study aimed to identify lncRNA molecules associated with copper-induced apoptosis. By examining the co-expression of lncRNA and genes related to copper-induced apoptosis, six lncRNA molecules were identified as having prognostic significance. This was determined through the use of univariate and multivariable Cox regression, survival analysis, risk mapping, ROC curve analysis, and heat mapping. The results demonstrated that these six lncRNA molecules accurately distinguished between high-risk and low-risk patients and reliably predicted prognosis in UCEC patients. Importantly, these prognostic factors were found to be independent of other commonly used clinical features.NRAV, AC026202.2 and AC090617.5 were protective indicators, while LINC01545, AL450384.1 and AC079466.2 were high-risk indicators. The novel biotargeted molecule lncRNA has the potential to treat cancer (Yao et al. 2022). Several lines of evidence indicate that lncRNA NRAV is involved in the immunological reaction against viruses and is a negative regulator of the antiviral response (Li et al. 2020; Zhang et al. 2018). According to research by Wang et al.,NRAV influences the Wnt/*β*-catenin signaling pathway to cells of hepatocellular carcinoma proliferation and invasion (Peng et al. 2018).Moreover, it has been shown thatNRAV is also a biomarker for clinical prognosis in HCC and low-grade gliomas (Feng et al. 2021; Maimaiti et al. 2021; Xu et al. 2021) LINC01545 is a long interstromal non-coding RNA that has been studied as a biomarker for predicting the development of diffuse large *B*-cell lymphoma (DLBCL) (Qin et al. 2012).Moreover, recent studies have identified AC090617.5 as a predictive indicator for lung adenocarcinoma and non-small cell lung cancer (Yao et al. 2023), the conclusions concur with our findings, in which AC090617.5 also plays a protective role.So far, the functions and detailed molecular interaction mechanisms of the remaining three lncRNAs have not been further explained.To completely understand the roles performed through these lncRNAs, further research is therefore required.

We adopted GO and KEGG enriched analysis investigated the differences in gene expression between the low and high categories to better understand the molecular mechanisms underlying endometrial cancer-related genes. The findings indicate that the molecular function enrichment of DEGs is primarily concentrated on extracellular structural organization and external encapsulated structural organization.The main manifestations of molecular function enrichment are signaling receptor activator, receptor ligand activity, and sulfur compound binding, the main cellular component is the type I collagen containing extracellular matrix KEGG analysis showed that DEGs in neurodegeneration-a variety of disease pathways, muscle atrophy-lateral sclerosis, neuroactive ligand receptor interaction and other molecular pathways. Tumor cells, stromal cells, and extracellular matrix make up the majority of the TME (ECM). Tumor cells are involved in tumor progression, which is strongly related to other TME components (Pitt et al. 2016), especially the immune cells. And tumor patients for immunotherapy responsiveness, can be evaluated by TMB, TMB reflects the variation of tumor cell genome, high TMB (TMB-H) tumor patients have the potential to get more new antigen, and is related to tumor heterogeneity, theoretically high TMB can enhance tumor immunogenicity and reaction with ICI. Numerous studies have established that a high TMB is linked to a significant benefit from immunotherapy. According to our results, TMB was more prevalent in low-risk score group, and the survival study using TMB data and risk stratification demonstrated that people with high mutation load and low risk score had the best prognosis. When DNA mismatch repair (mismatch repair, MMR) function abnormal, microsatellite replication errors are not corrected and accumulated, makes the microsatellite sequence length or base composition changes, called microsatellite instability (microsatellite instability, MSI), at the same time can lead to genome present high mutation phenotype, and the study found that MSI is a predictor of immunotherapy efficacy in advanced solid tumors. The proportion of microsatellite height instability (MSI-high, MSI-H) was significantly higher in the low-risk category (41% VS 23%) than in the high-risk category, however according our research results. This research backs up our hypothesis that people with the disease with low risk will respond positively to immunotherapy and benefit more from it.

Additionally, we performed a susceptibility analysis on individuals with various risk scores (Zhang et al. 2022), and in the end, we discovered a connection between five chemotherapy medications and the risk score, 5-fluorouracil (5-FU) and PHA-665752 were connected with the lower risk score.Compared to the lower risk category, the greater risk category had higher IC50 values, and the other three drugs’ IC50-value, MG-132, MS-275 (Entinostat), Bortezomib was linked to a lower risk score, and in the low-risk group, the IC50 value was lower than it was in the high-risk group.5-FU is the first antimetabolizing drug synthesized according to certain assumptions, and it is the most widely used anti-pyrimidine drug clinically. It has good efficacy on digestive tract cancer and other solid tumors, and is crucial in the management of medical oncology. A new target for many solid cancers is the c-Met receptor tyrosine kinase (including lung cancer). PHA665752 in mouse xenografts from small cell (NCI-H441 and A549) and non-small cell (NCI-H441 and A549) lung cancer cell lines(NCI-H69), angiogenic conversion was caused by in of c-Met phosphorylation and angiogenesis at the c-Cbl binding site, due to this, thrombsinin-1 synthesis increased whereas vascular endothelial growth factor production dropped (Crosswell et al. 2009).These investigations indicate the effectiveness of competitive small-molecule ATP inhibitors in selective c-Met targeting, and they propose that PHA665752 could offer a potential tumor therapeutic approach. Bortezomib and MG-132 both belong to the proteasome inhibitor class of drugs, which are currently used in the treatment of various tumor diseases, and have achieved outstanding clinical effects. According to numerous studies, bortezomib inhibits the expansion of tumor cells by influencing the proteins associated to apoptosis, such as nuclear transcription factors, intra-cell apoptosis signals, and cell-cycle-related proteins. While Entinostat is a well-tolerated HDAC inhibitor and has shown its therapeutic potential in both solid and hematological tumors. Despite the fact that the effect of some medicines in UCEC has not been studied, our findings might offer fresh suggestions for their treatment.

There have been several previous reports on the establishment of cuproptosis-related lncRNA models for predicting the prognosis of UCEC (Hu et al., 2023; Qi et al. 2023). Compared to a study by Qi et al., our model incorporated new lncRNAs (LINC01545, NRAV, AL450384.1, AC079466.2 and AC090617.5), and we further explored differential expression of lncRNAs in the model as well as CCLE database. Overall, the actual survival and predicted survival of the nomogram in our signature showed good consistency, as indicated by calibration curves. When evaluating survival predictions, AUCs at 1, 3 and 5 year were 0.778, 0.810 and 0.854 for the training group, respectively, which were significantly higher than those of previous studies.Our signature was also valuable for predicting PFS in UCEC patients. In summary, our prognostic model has good and stable prognostic prediction ability.

Finally, the identification of regulatory linkages between mRNA, lncRNA, and miRNA in UCEC holds potential for advancing our understanding of the molecular mechanisms involved in UCEC formation. This knowledge can contribute to the improvement of diagnostic and therapeutic approaches for UCEC, as well as the creation of tailored pharmaceutical interventions (Zhou et al. 2023). The first step in exploring ceRNA is finding miRNA that may bind lncRNA.Therefore, we predicted the downstream target genes of CRLs and visualized the results using the Cytoscape program (Fig.S?).The results showed that AL450384.1 and AC079466.2 may be regulatory hubs, AL450384.1 may exert a biological function by regulating downstream microRNA-181.Previous studies have shown that the miR-181 family is dysregulated in multiple tumor tissues,and plays a key role in carcinogenesis, Rezaei et al. believe that microRNA-181 plays a dual role in the development of human cancer (Rezaei et al. 2020).In addition,our data also reveal a substantial correlation of CRLs with miR-3140, Feng, et al. found Hypoxia-induced circCCDC66 promotes the tumorigenesis of colorectal cancer via the miR-3140/autophagy pathway (Feng et al. 2020) which may open up new UCEC research avenues.However, the impact of these pathways on tumor formation requires further validation through foundational tests.

Furthermore,some limitations of our study remain. For example, more clinical groups may be needed to cross-verify the prognostic accuracy and predictability of the characteristic. Furthermore, additional research is required to understand the mechanisms underlying the association as between cuproptosis-related lncRNAs and the prognosis of the UCEC.

## Conclusions

In conclusion,our research establishes a novel predictive model with six genes and speculate that cuproptosis may play a role in the development of UCEC. And we find that CRLs are significantly associated with abnormal immune infiltration expression and may be important genes for immune regulation of UCEC. Moreover, CRLs gene has a high predictive value for the prognostic risk of UCEC patients and can provide a reference for guiding immunotherapy in UCEC patients.

## Supplementary Information

Below is the link to the electronic supplementary material.

Supplementary file1 (DOCX 2395 KB)— Supplementary Table 1.19 cuproptosis-related genes (CRGs) were acquired from TCGA and previous publications. Supplementary Table 2.Clinicopathologic characteristics in entire, testing, and training groups.Supplementary Table3.The sequences of primers used in our study.FIG.S3 Train,test and all cohort clinical characteristics and risk score ROC curves.Fig.S8-A Results of immune cell infiltration under the TIMER algorithm.Fig.S8-B The relationship between immune cell infiltration analysis using the CIBERSORT algorithm and CRLs score.Fig.S12 CCLE database validation.Fig.S13 Network interaction regulation of CRLs and miRNA composition.

## Data Availability

The data used to support the findings of this study are available from the corresponding author upon request.

## Abbreviations

UCEC: Uterine corpus endometrial carcinoma
CRLs: Cuproptosis-related lncRNAs
LASSO: Least absolute shrinkage and selection operator
TCGA: The Cancer Genome Atlas
DEGs: Differentially expressed genes
OS: Overall survival
ROC: Receiver operating characteristic
GO: Gene Ontology
KEGG: Kyoto Encyclopedia of Genes and Genomes
PCA: Principal component analysis
AUC: Area under the curve
HR: Hazard ratio
GDSC: Genomics of Drug Sensitivity in Cancer
TME: Tumor Microenvironment
IC50: Half-maximal inhibitory concentration
