# Supplementary material for: Cuproptosis-Associated lncRNA Gene Signature Establishes New Prognostic Profile and Predicts Immunotherapy Response in Endometrial Carcinoma
Source: Biochem Genet. 2023 Dec 18;62(5):3439–66. doi: 10.1007/s10528-023-10574-8 (PMC11427535; doi:10.1007/s10528-023-10574-8)
Supplement: Supplementary file 1 — Supplementary file1 (DOCX 2395 KB)— Supplementary Table 1.19 cuproptosis-related genes (CRGs) were acquired from TCGA and previous publications. Supplementary Table 2.Clinicopathologic characteristics in entire, testing, and training groups.Supplementary Table3.The sequences of primers used in our study.FIG.S3 Train,test and all cohort clinical characteristics and risk score ROC curves.Fig.S8-A Results of immune cell infiltration under the TIMER algorithm.Fig.S8-B The relationship between immune cell infiltration analysis using the CIBERSORT algorithm and CRLs score.Fig.S12 CCLE database validation.Fig.S13 Network interaction regulation of CRLs and miRNA composition. [file 10528_2023_10574_MOESM1_ESM.docx]

**Supplementary Material**

**Supplementary Table 1**

| **NFE2L2**  **NLRP3**  **ATP7B**  **ATP7A**  **SLC31A1**  **FDX1**  **LIAS**  **LIPT1**  **LIPT2**  **DLD**  **DLAT**  **PDHA1**  **PDHB**  **MTF1**  **GLS**  **CDKN2A**  **DBT**  **GCSH**  **DLST** |
| --- |

19 cuproptosis-related genes (CRGs) were acquired from TCGA and previous publications.

| **Covariates** | **Type** | **Total** | **Test** | **Train** | **Pvalue** |
| --- | --- | --- | --- | --- | --- |
| Age | <=65 | 306(56.56%) | 156(57.56%) | 150(55.56%) | 0.7005 |
|  | >65 | 235(43.44%) | 115(42.44%) | 120(44.44%) |  |
| Stage | I | 339(62.43%) | 166(61.25%) | 173(63.6%) | 0.6062 |
|  | II | 52(9.58%) | 26(9.59%) | 26(9.56%) |  |
|  | III | 123(22.65%) | 61(22.51%) | 62(22.79%) |  |
|  | IV | 29(5.34%) | 18(6.64%) | 11(4.04%) |  |
| Grade | G1 | 99(18.23%) | 51(18.82%) | 48(17.65%) | 0.5723 |
|  | G2 | 121(22.28%) | 66(24.35%) | 55(20.22%) |  |
|  | G3 | 312(57.46%) | 148(54.61%) | 164(60.29%) |  |
|  | G4 | 11(2.03%) | 6(2.21%) | 5(1.84%) |  |

**Supplementary Table 2**

Clinicopathologic characteristics in entire, testing, and training groups.

| **Primers name** | Sequences of primers (5'→3') |
| --- | --- |
| H-GAPDH-F | GGAGCGAGATCCCTCCAAAAT |
| H-GAPDH-R | GGCTGTTGTCATACTTCTCATGG |
| H-AC026202.2-F | GCAGTTCCTCCTTCCCACTC |
| H-AC026202.2-R | GTGTTAGCCAGGGTGGTCTC |
| H-NRAV-F | GGAGTTGATGCCTCCGAACA |
| H-NRAV-R | ATGACCGGAGCTGAAAGGTG |
| H-LINC01545-F | TGGGATTACAAGCATGAGC |
| H-LINC01545-R | CAGAAGGATGGGAGGGTGG |
| H-AL450384.1-F | CCACAATCTCCTCCACCTC |
| H-AL450384.1-R | CCTGTAATGCCAGCACTTT |
| H-AC079466.2-F | CCTGTCAGTGGCAGAAGTT |
| H-AC079466.2-R | CTTAGGGAAACCAAAGCAG |
| H-AC090617.5-F | CGAGCCATTAGACTTTAGC |
| H-AC090617.5-R | GTGCCACTTACAGAAACAA |

**Supplementary Table3**

The sequences of primers used in our study were as follows.


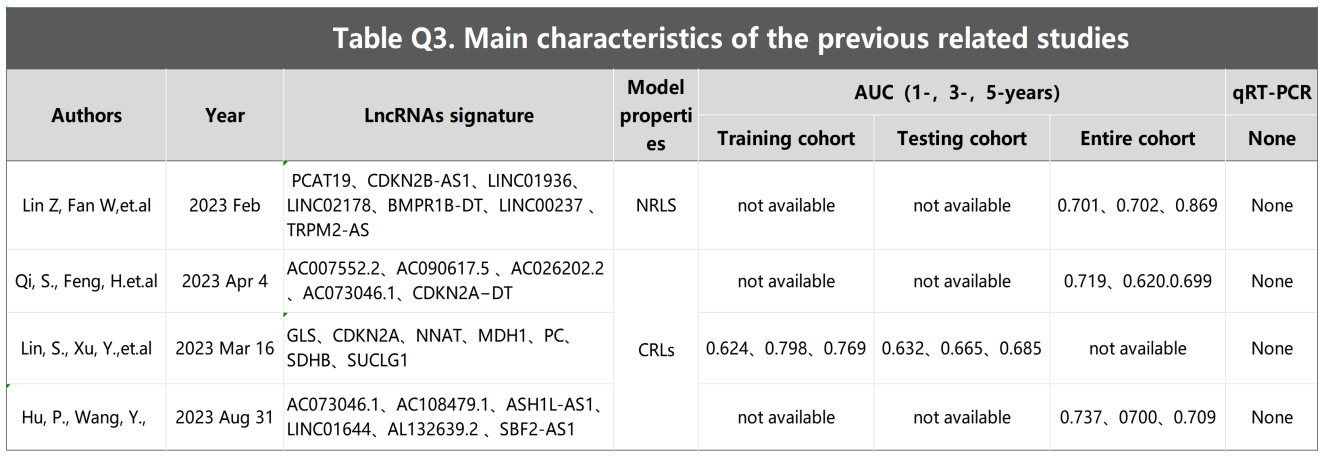


**Supplementary Table Q3**

Table Q3. Main characteristics of the previous related studies.


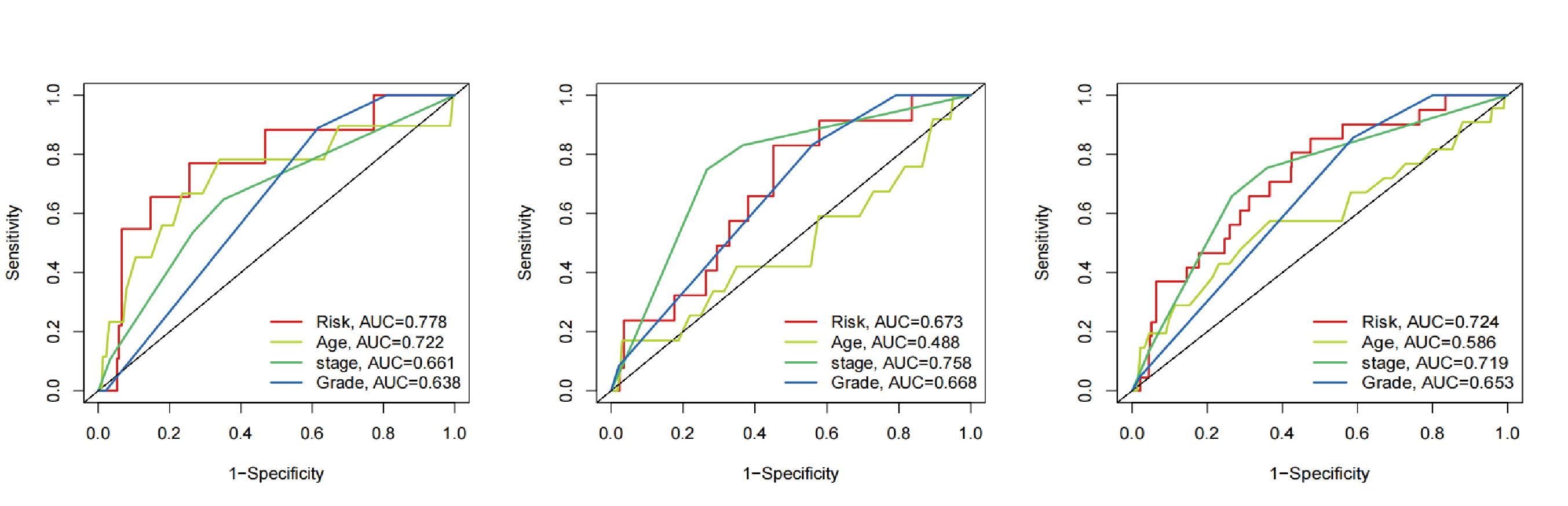


**FIG.S3** Train,test and all cohort clinical characteristics and risk score ROC curves.


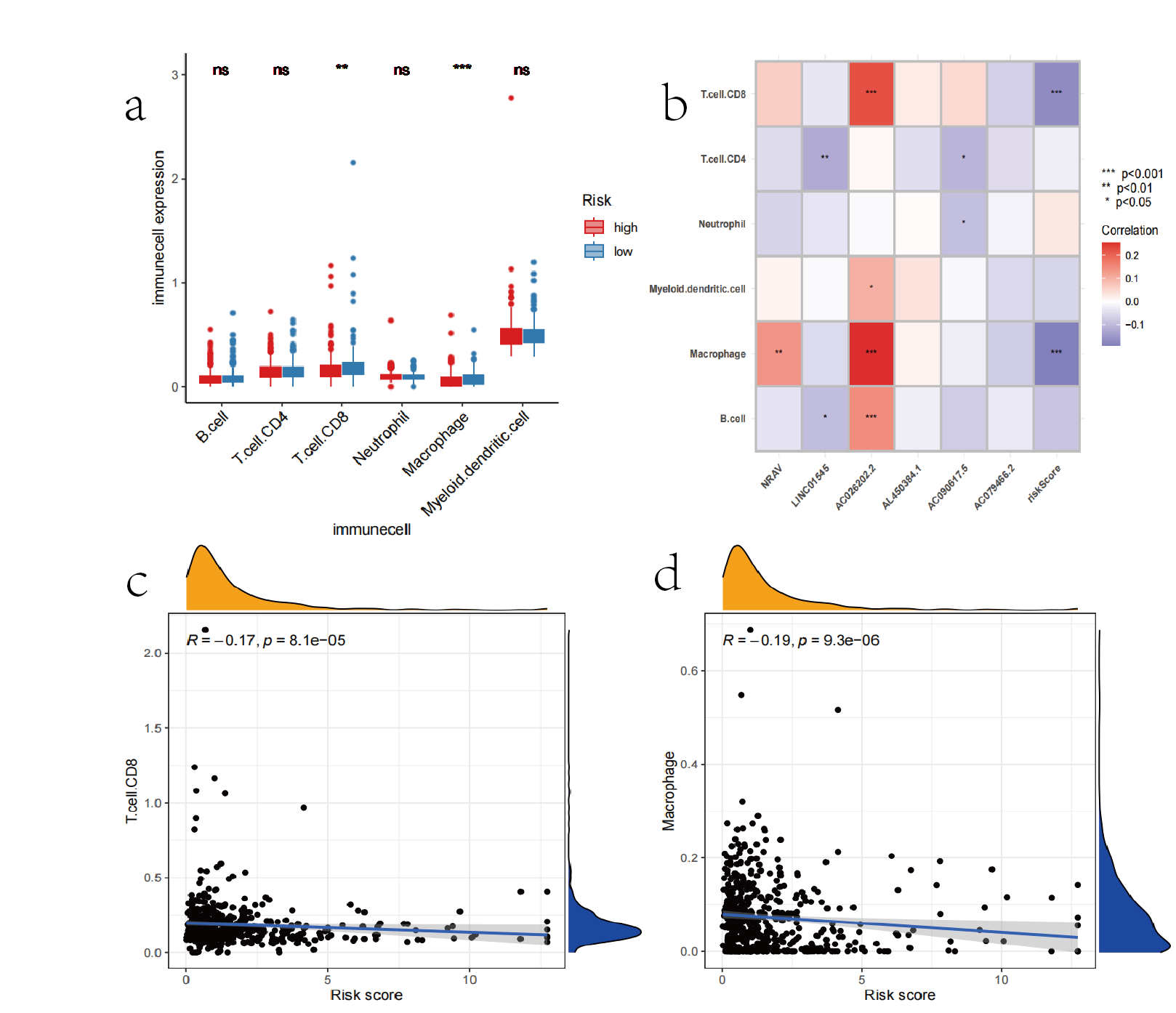


**Fig.S8-A** Results of immune cell infiltration under the TIMER algorithm.（a）Boxplot of the differential results of six immune cell infiltrates in the high and low risk groups.(b) Heatmap of correlation between six immune cells and CRLs and their risk scores.(c-d)Correlation analysis of the degree immune infiltration and the risk score(T cell CD8 + _TIMER , Macrophage _ TIMER ).

**
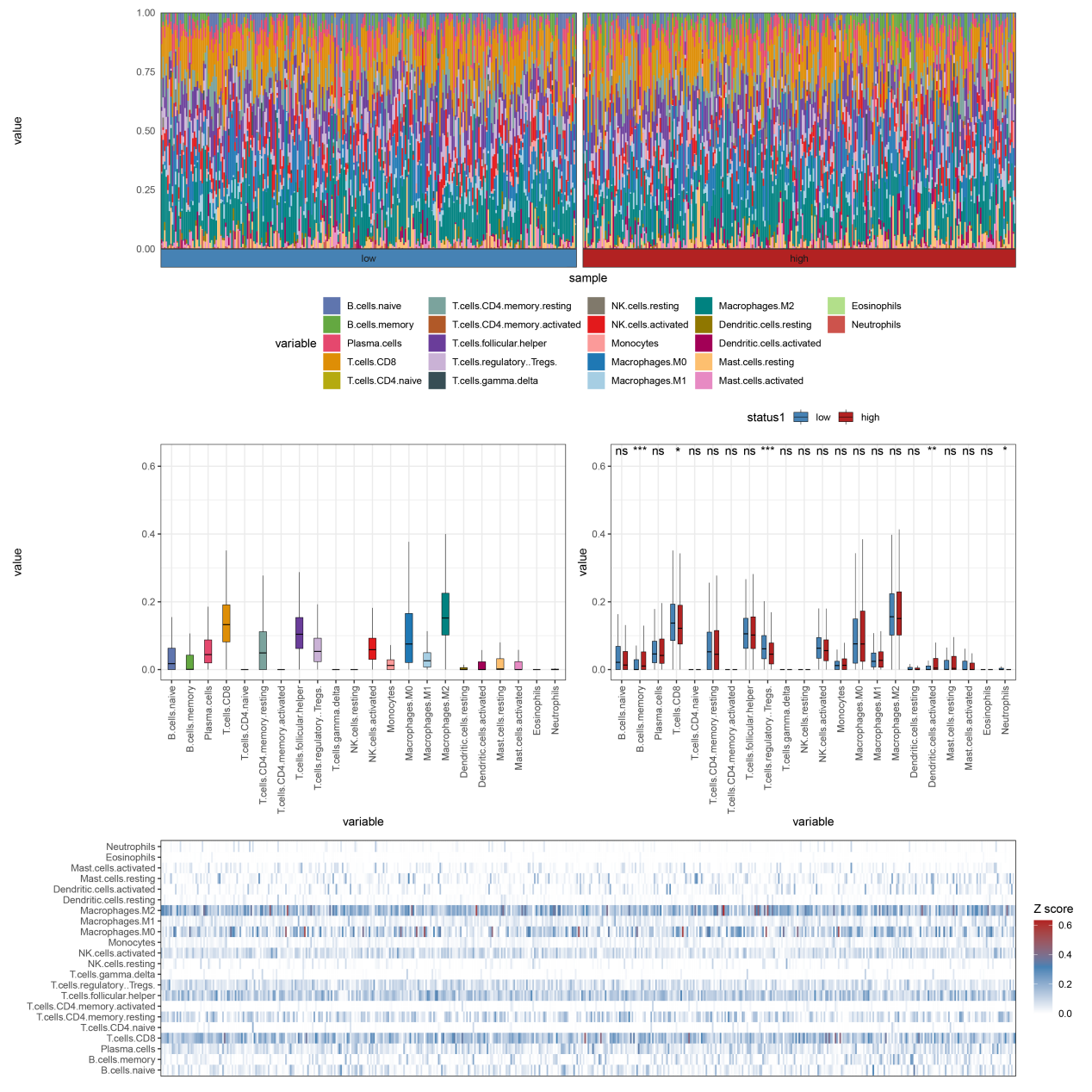
**

**Fig.S8-B** The relationship between immune cell infiltration analysis using the CIBERSORT algorithm and CRLs score.


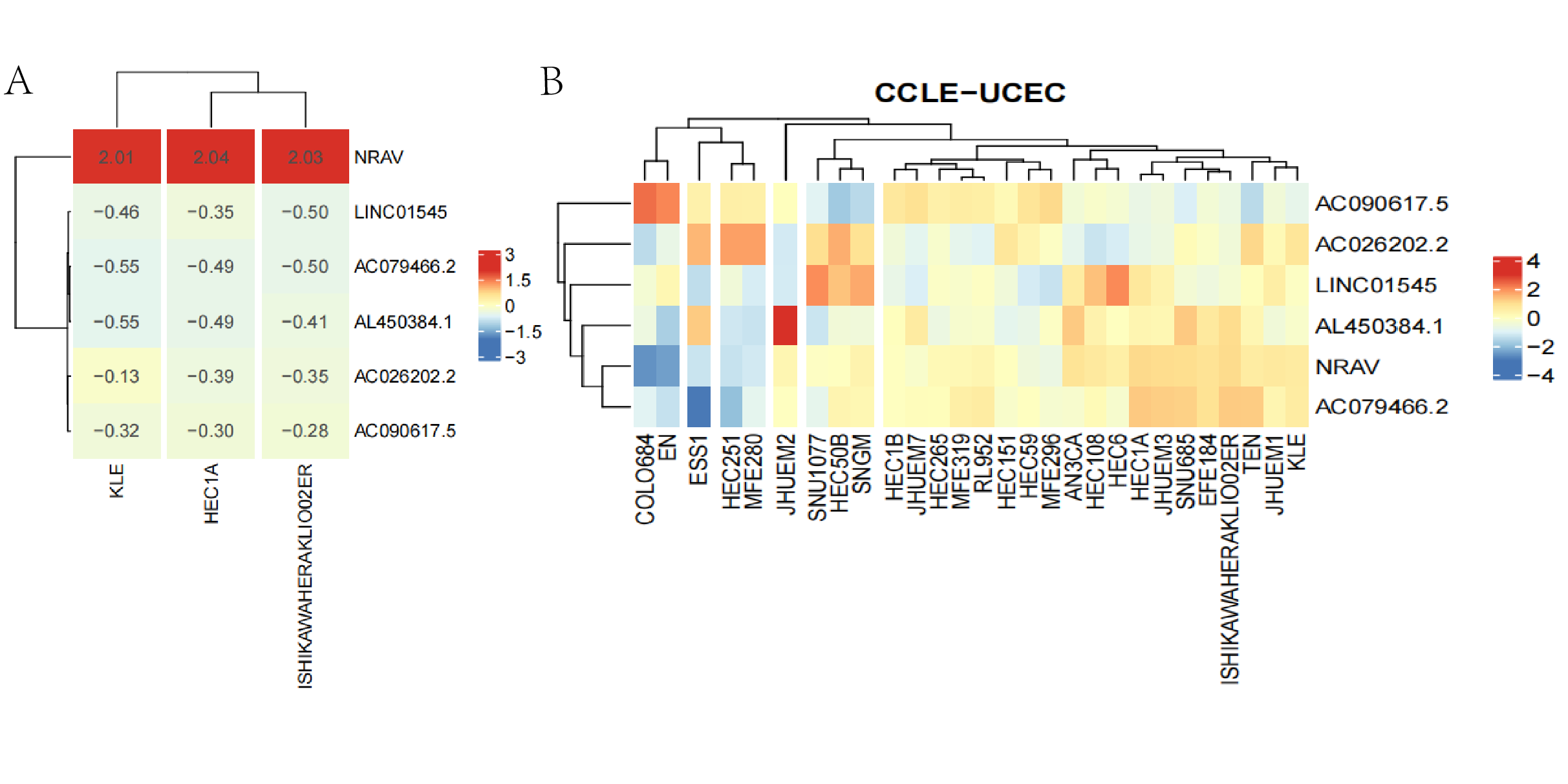


**Fig.S12 CCLE database validation.(A)** Heat maps of the expression levels of the three cell lines(KLE, HEC-1A, IshikawaHER) ,the numbers represent the degree of expression rather than the expression level after clustering.**(B)** Heat map of six lncRNAs expression in 28 endometrial cancer cell lines, obtained from the CCLE database.


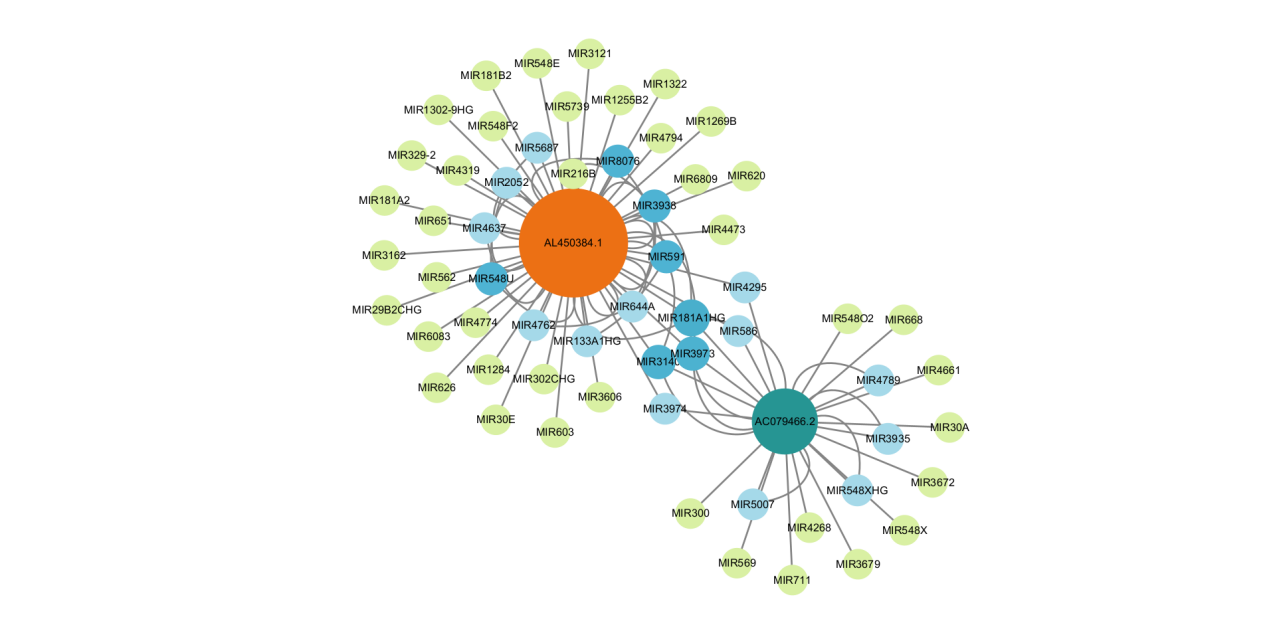


**Fig.S13** Network interaction regulation of CRLs and miRNA composition.
